# Cost-effectiveness analysis of different types of human papillomavirus vaccination combined with a cervical cancer screening program in mainland China

**DOI:** 10.1186/s12879-017-2592-5

**Published:** 2017-07-18

**Authors:** Xiuting Mo, Ruoyan Gai Tobe, Lijie Wang, Xianchen Liu, Bin Wu, Huiwen Luo, Chie Nagata, Rintaro Mori, Takeo Nakayama

**Affiliations:** 1 Department of Health Informatics, Kyoto University School of Public Health, Kyoto, Japan; 2 Department of Health Policy, National Center for Child Health and Development, Okura 2-10-1, Setagaya-ku, Tokyo, 157-8535 Japan; 3 Department of Gynecology, Qilu Hospital of Shandong University, Jinan, China; 4 College of Pharmacy, The University of Tennessee Health Science Center, Memphis, TN USA; 5 Medical Decision and Economic Group, Department of Pharmacy, Ren Ji Hospital affiliated with Medical School of Shanghai Jiao tong University, Shanghai, China; 6 Nanjing Drum Tower Hospital affiliated Medical School of Nanjing University, Nanjing, China; 7 Department of Education for Clinical Research, National Centre for Child Health and Development, Tokyo, Japan

**Keywords:** Cervical cancer, Cervical intraepithelial neoplasia, Incremental cost-effectiveness ratio, Vaccine

## Abstract

**Background:**

China has a high prevalence of human papillomavirus (HPV) and a consequently high burden of disease with respect to cervical cancer. The HPV vaccine has proved to be effective in preventing cervical cancer and is now a part of routine immunization programs worldwide. It has also proved to be cost effective. This study aimed to assess the cost-effectiveness of 2-, 4-, and 9-valent HPV vaccines (hereafter, HPV2, 4 or 9) combined with current screening strategies in China.

**Methods:**

A Markov model was developed for a cohort of 100,000 HPV-free girls to simulate the natural history to HPV infection. Three recommended screening methods (1. liquid-based cytology test + HPV DNA test; 2. pap smear cytology test + HPV DNA test; 3. visual inspection with acetic acid) and three types of HPV vaccination program (HPV2/4/9) were incorporated into 15 intervention options, and the incremental cost-effectiveness ratio (ICER) was calculated to determine the dominant strategies. Costs, transition probabilities and utilities were obtained from a review of the literature and national databases. One-way sensitivity analyses and threshold analyses were performed for key variables in different vaccination scenarios.

**Results:**

HPV9 combined with screening showed the highest health impact in terms of reducing HPV-related diseases and increasing the number of quality-adjusted life years (QALYs). Under the current thresholds of willingness to pay (WTP, 3 times the per capita GDP or USD$ 23,880), HPV4/9 proved highly cost effective, while HPV2 combined with screening cost more and was less cost effective. Only when screening coverage increased to 60% ~ 70% did the HPV2 and screening combination strategy become economically feasible.

**Conclusions:**

The combination of the HPV4/9 vaccine with current screening strategies for adolescent girls was highly cost-effective and had a significant impact on reducing the HPV infection-related disease burden in Mainland China.

**Electronic supplementary material:**

The online version of this article (doi:10.1186/s12879-017-2592-5) contains supplementary material, which is available to authorized users.

## Background

Cervical cancer is one of the most widespread gynaecological cancers worldwide and remains the second leading cause of gynaecological-related mortality. More than 85% of cases occur in developing countries, resulting in an estimated 275,000 deaths annually [1]. In mainland China, despite its decreasing incidence in recent years, cervical cancer remains among the top ten most common malignancies among women and is a leading cause of cancer-related mortality nationwide [2, 3]. Human papillomavirus (HPV) infection is the main cause of cervical cancer and can be detected in more than 95% of uterine carcinomas. Vaccinations are effective in preventing HPV infection, and a number of vaccine options have recently become available. The 2-valent HPV vaccine (HPV2), which targets HPV 16/18 and the 4-valent HPV vaccine (HPV4), which targets 6/11/16/18, have been approved in more than 100 countries worldwide. Both vaccines were highly effective in clinical trials and shown to be cost effective in health economic studies in various countries [4]. The 9-valent HPV vaccine (HPV9) targeting five additional oncogenic HPV types (31, 33, 45, 52, and 58) improved protection against 90% of cervical cancers, an increase of 20% from the previous figure, and has been licensed in more than 30 countries [5, 6]. In July 2016, a decade after international approval of the first HPV vaccine, the Chinese government approved GSK Cervarix™, a 2-valent HPV vaccine expected to become commercially available in early 2017 [7, 8]. In addition, some other HPV-related cancers (such as oropharyngeal, anal, and vulvar cancer) caused by HPV 16/18/31/33/45/52/58 may be prevented by the vaccines [9–11].

Further, as recommended by the World Health Organization (WHO), screening is a common preventative strategy targeting women of reproductive age for the early detection and treatment of HPV infection, cervical cancer, and cervical intraepithelial neoplasia (CIN). In developing countries, routine screening programs reportedly reduced the incidence of cervical cancer by up to 60% [12]. In 2005 the Ministry of Health of China formed guidelines for the screening, early detection, and treatment of cervical cancer [13]. The target demographic of these guidelines was women older than 21 years and those who engaged in sexual activity for more than three years. The guidelines recommend three different protocols each designed for a particular socioeconomic stratum: 1) primary screening by liquid-based cytology test + HPV DNA test, which has optimal sensitivity and specificity but is expensive and requires well-equipped infrastructure and ample resources; 2) primary screening by pap smear cytology test + HPV DNA test, requiring less infrastructure and resources; and 3) primary screening by visual inspection with acetic acid (VIA), a basic protocol designed for low income settings. Although a government-sponsored VIA and cytology screening program has been carried out in some regions [14], no national cervical screening program currently exists on mainland China. According to some local surveys, the coverage rate of current screening programs is approximately 10 ~ 30% [15, 16] and even when offered free of charge, remained at around 50% [17].

Although the cost-effectiveness of the HPV vaccine in China has been analysed [18–20], a thorough assessment specific to China of HPV2, 4, and 9 and the three previously mentioned screening protocols still remains to be done. Therefore, this study aims to evaluate the cost-effectiveness of different HPV vaccinations as alternatives, and as an adjunct to the three primary screening strategies currently in use in mainland China, with consideration of diversified geographical characteristics.

## Methods

### Overview of the model

**Fig. 1:**
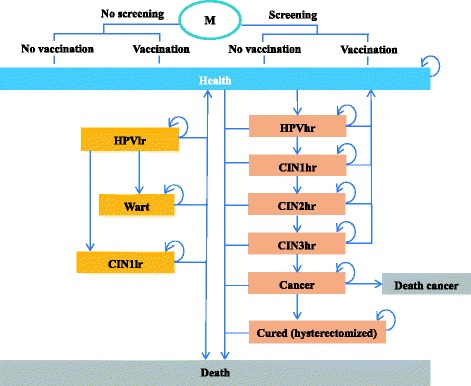
Markov model of the history of high and low risk type of HPV. The *arrows* direct transitions from one state to another. hr, high-risk; lr, low-risk; CIN, cervical intraepithelial neoplasia; HPV, human papillomavirus

A Markov model was used to simulate the natural history of HPV infection and to estimate the economic consequences of HPV-related diseases from a societal perspective view (Fig. 1). The rationale for using the model was the example provided by several previous natural history models of HPV simulating high and low risk HPV infections separately. In this study, the model was adapted to the Chinese context (three different screening strategies and diagnosis/treatment flow specific to the Chinese setting) in order to reflect local screening and treatment practices [21–23].

We assumed that girls under 12 years old were virgins. In our analysis, we simulated a cohort of 100,000 HPV-free girls (from 12 years old and followed up until death). In the strategies using vaccination, individuals were given the respective vaccines at the beginning of the simulation. HPV genotypes were divided into high risk (carcinogenic HPV 16/18/31/33/45/52/58, etc.) and low risk (HPV 6/11 and etc.) groups in the analysis. Health states were defined based on the natural history of HPV infection in the following manner: healthy, low risk HPV infection, high risk HPV infection, mild cervical intraepithelial neoplasia (CIN 1) caused by high risk HPV infection, moderate cervical intraepithelial neoplasia (CIN 2) caused by high risk HPV infection, severe cervical intraepithelial neoplasia or carcinoma in situ (CIN 3) caused by high risk HPV infection, CIN1/2 caused by low risk HPV infection, genital warts, invasive cervical cancer (ICC) (stages I-IV as defined by the International Federation of Gynaecology and Obstetrics, FIGO), cervical cancer survival, death from cervical cancer, and death from other causes [21]. Oropharyngeal, anal, and vulvar cancers were not included in this study because differing proportions of these cancers are caused by HPV, and it is difficult to quantify the effects of vaccination on the incidence of HPV caused by the strains covered. Furthermore, the epidemiological data on HPV-related cancers other than cervical cancer caused by HPV 16/18 or HPV 16/18/31/33/45/52/58 were not available in China. Since the impact of the HPV vaccines on other non-cervical HPV-related cancers have not been included in the analysis, the projected health benefits are likely underestimated.

The Markov model developed for this study comprehensively assessed relevant and currently in-use preventative strategies including both vaccination and screening in China. The screening strategies included the three protocols recommended in the *2005 Guidelines for Screening, Early Detection and Treatment of Cervical Cancer* [13]: 1) liquid-based cytology test + HPV DNA test; 2) pap smear cytology test + HPV DNA test; 3) visual inspection with acetic acid (VIA) and a colposcopy following a positive result to make a definite diagnosis. The vaccination options were HPV2 (CERVARIX,HPV4 (GARDASIL®) and the newly developed HPV9 (GARDASIL®9). We compared 15 preventative strategies consisting of either a single modality or a combination of vaccination and screening, namely, Screening 1, Screening 2, Screening 3, HPV2, HPV4, HPV9, Screening 1 + HPV2, Screening 2 + HPV2, Screening 3 + HPV2, Screening 1 + HPV4, Screening 2 + HPV4, Screening 3 + HPV4, Screening 1 + HPV9, Screening 2 + HPV9, Screening 3 + HPV9, and no intervention.

### Screening strategies

The target population for screening was women older than 21 years. Screening every three years was recommended. Based on the cervical cancer prevention guidelines of mainland China and several Chinese reports, we assumed that a liquid-based cytology test would have a sensitivity of 0.85 and a specificity of 0.90 [24]. The HPV DNA test had a sensitivity of 0.95 and specificity of 0.85 [24]. The pap smear had a sensitivity of 0.65 and specificity of 0.88 [24]. The VIA test had a sensitivity of 0.68 and specificity of 0.85 [24–26]. We assumed that the colposcopy + biopsy combination would have perfect sensitivity and specificity for the detection of CIN2+ [27].

A screening coverage rate of 20% was assumed due to the large geographical diversity in socioeconomic development, and 10%–100% was set for the sensitivity analysis [28].

### Vaccine characteristics

As vaccine efficacy data specific to the Chinese population were unavailable, the data used here were obtained by multiplying the direct efficacy targeting specific HPV types by serotype coverage on mainland China. In the clinical trials, HPV2 (CERVARIX),HPV4 (GARDASIL®), and HPV9 (GARDASIL®9) all showed excellent efficacy against the relevant CIN and AIS (Adenocarcinoma in situ) in women with HPV DNA negative for oncogenic HPV types at baseline: HPV2: 96.5% (95% CI: 91.6%–98.9%) [29]; HPV4: 96.0% (95% CI: 92.3%–98.2%) [10]; HPV9: 96.0% (95% CI: 92.3%–98.2%) for HPV 6, 11, 16, or 18-related CIN or AIS, and 96.7% (95% CI: 80.9%–99.8%) for the additional five HPV types, namely, HPV 31, 33, 45, 52, and 58 [11]. The efficacy of the HPV4 and HPV9 against specific HPV-related genital warts was 99.0% (95% CI: 96.2%–99.9%) [11]. HPV2 was assumed to have the same efficacy against genital warts (99.0%, 95% CI: 96.2%–99.9%). The parameter: HPV serotype coverage of each vaccine, was obtained from a doctoral dissertation, an epidemic research on HPV distribution in genital warts, ICC and CIN2/3 samples from 18 hospitals in 7 geographic regions [30]. HPV6, 11 and 16 distribution in genital warts patients were not significantly differed by urban and rural areas. Nor did HPV prevalence in squamous cell carcinoma patients differ notably by region [30]. The following are the HPV types with their respective incidence for CIN2/3: HPV 6: 1.26%; HPV 16: 75.94%; HPV 11: 0; HPV 18: 7.70%; HPV 31: 3.14%; HPV 33: 0.94%; HPV 45: 1.10%; HPV 52: 2.20%; and HPV 58: 2.20% [30]. The rates for genital warts were HPV 6: 46.6%; HPV 11: 42.4%, and HPV 16: 11.8% [30]. The parameters for the sensitivity analysis were the baseline value ±25%.

After calculation, the overall efficacy of HPV2, HPV4, and HPV9 against cervical cancer was 80.72% (57.47%–98.9%), 81.51% (58.78%–98.2%), and 90.78% (66.54%–100%), respectively, and that against genital warts was 10.36% (7.56%–13.07%), 86.06% (62.72%–99.99%) and 86.06% (62.72%–99.99%), respectively.

Moreover, considering the reported efficacy data and demonstration of immunological memory [31], we assumed that the protection conferred by the vaccines would be life-long and obviate the need for a booster. Because of the relatively low coverage of the current prevention program, cross protective effects were not taken into account.

A vaccine coverage rate was assumed as 20%, and 10%–100% was set for the sensitivity analysis.

### Transition probabilities

Each strategy (screening plus vaccination or screening only) modelled probabilities for mutually exclusive health states within a one-year cycle. At each transition, the model produced figures for the costs incurred and the QALYs according to the individual’s health condition. Each year, the target population was exposed to an age-specific risk of HPV infection that could persist, progress (to CIN 1, CIN 2–3, genital warts, etc.) or resolve. In those who developed CIN 3, their disease might persist, remit, or progress to localized (FIGO stage I and IIA), regional (FIGO IIB to IVA) or metastatic invasive cancer (FIGO IVB). Given the course of cervical cancer, each individual might continue to suffer from the disease, die, or experience complete remission. Each year individuals faced age-specific risks of dying from other causes or of undergoing a hysterectomy for reasons unrelated to cervical neoplasia. Women who underwent a hysterectomy did not develop cervical cancer in our model. Table 1 summarizes the initial value and all the transition probabilities derived from the previous reports and research projects of the Cancer Institute and Hospital, Chinese Academy of Medical Sciences (CICAMS). Screening Technologies to Advance Rapid Testing for Cervical Cancer Prevention (START) was calibrated to reflect Chinese epidemiology [32].

**Table 1:** Model variables: Baseline values and ranges used in sensitivity analysis

Variable	Base case	Plausible range	References
Natural history of HPV	Age-specific table		
*HPV-2*	Serotypes covered*efficacy		
Efficacy for cervical cancer	0.836*0.965	0.575–0.989	[27, 30]
Efficacy for genital warts	0.107*0.990	0.756–0.131	Assumed
*HPV-4*			
Efficacy for cervical cancer	0.849*0.960	0.588–0.982	[28, 30]
Efficacy for genital warts	0.869*0.990	0.627–0.999	[29, 30]
*HPV-9*			
Efficacy for cervical cancer	0.849*0.960 + 0.096*0.967	0.665–1.000	[29, 30]
Efficacy for genital warts	0.869*0.990	0.627–0.999	[29, 30]
*Pap smear*			
Sensitivity	0.65	0.50–0.80	[22]
Specificity	0.88	0.85–0.90	[22]
*Liquid-based cytology test*			
Sensitivity	0.85	0.80–0.90	[22]
Specificity	0.90	0.85–0.95	[22]
*HPV DNA test*			
Sensitivity	0.95	0.80–0.98	[22]
Specificity	0.85	0.80–0.90	[22]
VIA			
Sensitivity	0.68	0.50–0.70	[22–24]
Specificity	0.85	0.66–0.96	[22–24]
*Colposcopy and biopsy*			
Sensitivity	1	0.50–1.0	[25]
Specificity	1	0.50–1.0	[25]
Age begin to screen	20	18–45	Assumed
Screening intervals	3	1,3,5,10	Assumed
Screening coverage	0.2	0.1–1.0	Assumed
Vaccine coverage	0.2	0.1–1.0	Assumed
*Costs (USD)*			
HPV 2/4 vaccine (3 does)	403.23	0.5X-1.5X	[38]
HPV 9 vaccine (3 does)	447.10	0.5X-1.5X	[38, 39]
vaccine administration (3 does)	4.84	0.5X-1.5X	Chinese Anti-Cancer Association
Pap smear	6.75	0.5X-1.5X	Chinese Anti-Cancer Association
Liquid-based cytology	43.89	0.5X-1.5X	Chinese Anti-Cancer Association
HPV DNA test	56.45	0.5X-1.5X	Chinese Anti-Cancer Association
VIA	5.06	0.5X-1.5X	Chinese Anti-Cancer Association
Colposcopy and biopsy	32.26	0.5X-1.5X	Local field study
Loop electrosurgical excision procedure (LEEP)	403.23	0.5X-1.5X	Local field study
Cold knife conisation	887.10	0.5X-1.5X	Local field study
Hysterectomy	2419.35	0.5X-1.5X	Local field study
Localized cancer	3225.81	0.5X-1.5X	Local field study
Regional cancer	4838.71	0.5X-1.5X	Local field study
Metastatic cancer	6451.61	0.5X-1.5X	Local field study
Genital warts	161.29	0.5X-1.5X	Local field study
*Utilities*			
CIN1	0.9965	0.992603–1.0	[33]
CIN2	0.984	0.876–1.0	[33]
CIN3	0.984	0.806–1.0	[33]
Cancer	0.693	0.56–0.76	[33]
Genital warts	0.827	0.701–0.933	[36]
Cancer survival	0.850	0.82–0.88	[26, 34, 35]
*Compliance of treatment*			
CIN2+	0.9		[26]
Cancer	1.0		Assumed
Choose hysterectomy when CIN3	0.2 when >35		[26]
Discount rate of cost	3%	0–6%	Assumed
Discount rate of effectiveness	3%	0–6%	Assumed

### Health utilities

Utilities are a measure of the quality of life rated on a scale of 0 (death) to 1 (optimal health), and are based on economic studies in China. CIN 2 was assigned a lower utility (0.88) [33], while CIN 3 was assigned an utility of 0.81 for a 1-year period [33]. In cases of ICC, a woman’s utility was assumed to decrease to 0.69 [33]. The utility of cervical cancer survivors was 0.85 [28, 34, 35]. The utility of patients with genital warts was determined to be 0.67 based on some Chinese studies [36, 37].

### Cost estimates

Cost estimates included the cost of vaccination, screening, and treatment of diseases related to HPV infection. Data were initially calculated in Chinese Yuan and then converted to US dollars in 2015 using the consumer price index (CPI) and the official exchange rate of the Chinese Yuan to US dollar (January 2015 exchange rate, USD$1 = CYN6.20). Although the three HPV vaccines mentioned above were not yet available on mainland China, *Gardasil,* a quadrivalent vaccine developed by Merck and *Cervarix,* a bivalent vaccine developed by GlaxoSmithKline*,* both costing about the same, were approved in Hong Kong. We estimated the costs of three doses according to the standard price in Hong Kong (2000 ~ 3000 CYN) [38], and the administration fee for each girl was estimated at USD$4.84 dollars. Cost of three doses of HPV9 vaccine was calculated by multiplying USD$ 403.23 by the ratio of cost of HPV4/HPV9 per dose ($147.78/$163.86 = 1.1088) [39]. Considering the likelihood that the costs on the mainland would be lower than in Hong Kong due to the lower economic status of the mainland population, the sensitivity analysis was performed with a value range of 0.5X-1.5X. Moreover, we approximated different screening costs using the current national tariff proposed by the Chinese Anti-Cancer Association [40]. The treatment costs were obtained from tertiary hospitals and the local health system. Treatment by FIGO staging was based on the recommendation of national and international guidelines [41]. A half cycle correction was used in order to achieve a closer approximation to proper reward/survival.

In terms of compliance, the baseline value for CIN 2/3 treatment extracted from Screening Technologies to Advance Rapid Testing for Cervical Cancer Prevention (START) was set at 90% [28]. The selection rate for a hysterectomy in the event of CIN 3 was set at 20% for those older than 35 years and 0% for all others [28]. When cervical cancer was detected or symptoms appeared, compliance with treatment was assumed to be 100%.

### Outcome measures

The model was programmed using TreeAge Pro 2011. We expressed the results in terms of the number of cases of cervical cancer, genital warts, and deaths from cervical cancer prevented, as well as the lifetime cost and QALYs gained. The incremental cost-effectiveness ratio (ICER) was calculated as incremental cost divided by the QALYs gained per woman by adding vaccination to the status quo (i.e., each of the three different screening strategies reflecting three different Chinese settings). According to the WHO, if the ICER is less than the per capita gross domestic product (GDP) a strategy can be considered as “very cost effective”, and if less than three times the per capita GDP, as “cost effective” [42]. Three times the 2015 per-capita GDP for China amounted to USD$23,879.52 [43]. An annual discount rate of 3% was applied to both costs and benefits.

### Sensitivity analyses

One-way sensitivity analysis was performed to explore the impact of the uncertainty of the parameters, taking into account simultaneous changes within the plausible ranges. The efficacy of each vaccine, the sensitivity and specificity of different tests, and the utilities associated with different health stages varied uniformly within the referred ranges. The cost parameters obtained varied uniformly in a range of +/− 50% of the deterministic estimate. The discount rate for cost and effectiveness varied uniformly between 0 to 6%. According to the results of tornado diagrams, threshold analyses were performed.

## Results

### Predicted cervical cancer mortality

Cervical cancer mortality in the trial (screening only without vaccination) was based on cancer statistics. The predicted cervical cancer mortality showed acceptable correspondence with data from the 2011 Chinese Cancer Registry Annual Report [44] (Additional file 1: Figure S1).

### Base case analysis

**Fig. 2:**
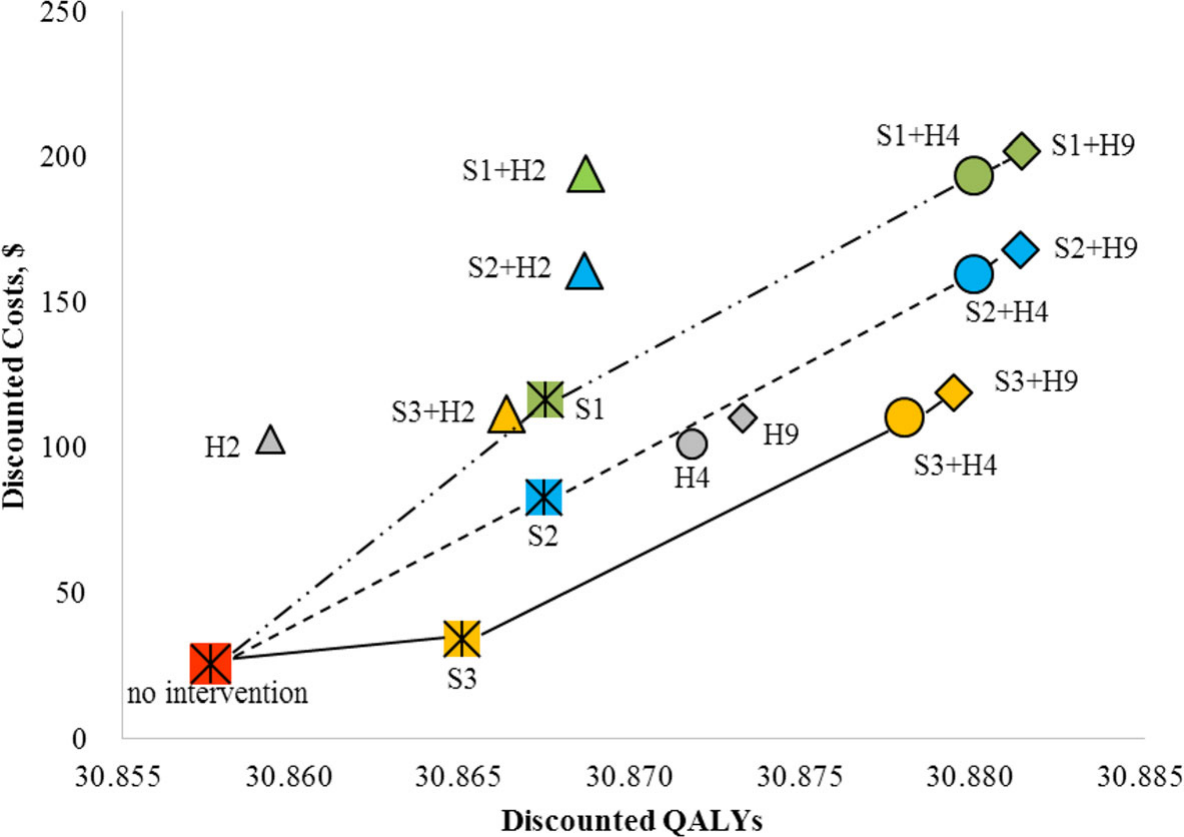
Comparing Discounted cost and QALYs in different screening settings. Three lines stand for three different screening settings. S is short for screening; H is short for HPV vaccine. *Green* dot means strategies with screening1, *blue* dot means strategies with screening2 and *yellow* dot means strategies with screening3

The cost-effectiveness analysis and the reduction in cervical cancer and warts for each strategy under base case assumptions based on the Monte Carlo simulation of 100,000 trials showed that among the 15 options consisting either of a single strategy or one combining vaccination and screening, HPV9 combined with screening yielded the highest number of QALYs. Screening 1 and Screening 2 and their respective combination strategies showed little difference in discounted QALYs but an obvious cost difference (Table 2). In the calculation of ICERs, we first compared each single strategy (vaccine or screening) with no intervention strategy. Among three different vaccines, HPV4 cost least and gained almost the same number of QALYs as HPV9, while HPV-2 was dominated by HPV-4 due to higher costs and lower effectiveness. Screening 3, on the other hand, cost least, gained nearly the same QALYs as the other two screenings, and showed the most cost-effectiveness, while Screen1 showed no cost-effectiveness due to its ICER is over 3 times WTP. Then we compared each combination strategy in three different screening settings. HPV2 in its respective combination strategies were always ruled out by absolute dominance (higher cost and lower effectiveness) and demonstrated no cost-effectiveness while the HPV4 and HPV9 combination strategies presented significant cost-effectiveness (each ICER was less than per capita GDP at 7960 USD). The pathway of the most cost-effective strategy at different levels of willingness-to-pay thresholds as determined by the ICERs in different screening settings demonstrated that the dominant strategy gradually shifted from screening only to screening + HPV4, and finally screening + HPV9 (Fig. 2). When competing all strategies against each other, Screening 3, Screening 3 + HPV4 and Screening 3 + HPV9 were cost-efficient with ICERs below the threshold WTP. Screening 2 + HPV9 and Screening 1 + HPV9 were also on the cost-effective frontier but were not cost-effective as their ICERs (USD$24,867/QALY; USD$1,162,147/QALY) was over WTP (3 times of per capita USD$23,880 USD) (Additional file 1: Figure S2).

**Table 2:** Cost-effectiveness analysis and reduction in cervical cancer and warts of each strategy under base-case assumptions based on Monte Carlo simulation of 100,000 trails

Strategy	Discounted costs	Discounted QALYs	IC/IE ($/QALYs)	Cancer reduction incidence (%)	Cancer mortality reduction (%)	HPVhr incidence reduction (%)	Warts incidence reduction (%)
No intervention	25.359	30.858	--	1893.000	348.000	895,739.000	100,676.000
*HPV-4* ^a^	101.631	30.872	5400.550^f^	15.850	18.200	18.130	15.570
*HPV-2* ^a^	103.205	30.859	Dominated	13.100	13.100	4.170	14.500
*HPV-9* ^a^	110.203	30.873	5768.350^f^	16.270	18.410	20.450	15.560
*Screen1* ^b, c^	116.544	30.867	959,735.859^h^ 9287.599^g^	18.860	19.020	10.400	5.900
Screen1 + HPV-4^c^	193.106	30.880	6070.026^f^	33.860	35.690	23.640	20.780
Screen1 + HPV-2^c^	194.301	30.869	Dominated	32.280	32.280	6.490	19.840
Screen1 + HPV-9^c^	201.724	30.881	6275.190^f^	34.390	35.950	25.820	20.800
*Screen2* ^b, d^	82.945	30.867	20,372.19^g^ 5886.421^f^	18.860	19.020	10.340	5.880
Screen2 + HPV-4^d^	159.397	30.880	6058.622^f^	33.860	35.690	23.610	20.730
Screen2 + HPV-2^d^	160.713	30.869	Dominated	32.280	32.280	6.460	19.790
Screen2 + HPV-9^d^	168.002	30.881	6262.947^f^	34.390	35.950	25.800	20.750
*Screen3* ^*b, e*^	34.004	30.865	1171.435^f^ 1171.435^f^	18.540	18.860	8.010	4.100
Screen3 + HPV-4^e^	110.341	30.878	5865.854^f^	32.750	35.130	21.650	19.220
Screen3 + HPV-2^e^	111.804	30.866	Dominated	31.170	31.720	3.890	18.280
Screen3 + HPV-9^e^	118.927	30.879	6098.722^f^	33.280	35.400	23.910	19.230

ICERs were calculated in 5 settings: ^a^no intervention, HPV-4, HPV-2 and HPV-9; ^b^no intervention, Screen3, Screen2 and Screen1; ^c^no intervention, Screen1, Screen1 + HPV-4, Screen1 + HPV-2, and Screen1 + HPV-9; ^d^no intervention, Screen2, Screen2 + HPV-4, Screen2 + HPV-2, and Screen2 + HPV-9; ^e^no intervention, Screen3, Screen3 + HPV-4, Screen3 + HPV-2, and Screen3 + HPV-9

^f^If 0 < ICER < per capita GDP (7960 USD), it is considered very cost effective; ^g^If per capita GDP (7960 USD) < ICER <3 times of per capita GDP (23,880 USD), it is considered as cost effective; ^h^If ICER >3 times of per capita GDP (23,880 USD), it is considered not cost effective. Absolute dominated: An option is said to be dominated if it both costs more and is less effective than a comparator

Based on the natural history (no intervention), 1893 cases got cervical cancer and 348 cases died of it. HPV and genital wart infections occurred 895,739 times and 100,676 times, respectively. Among all 15 strategies, Screening 1 + HPV9 showed the best preventative effect against HPV-related disease by reducing cervical cancer incidence by 34.39%, cancer mortality cases by 35.95%, HPV-Hr infection rate by 25.82%, and genital wart incidence by 20.80% compared to no intervention. Among the three screening protocols, Screenings 1 and 2 showed no significant difference in their preventative effects against diseases related to HPV infection but were superior to Screening 3. Among the three vaccines, HPV9 showed the strongest preventative effect against HPV-related disease compared to the other two in both the single and combination strategies (Table 2).

### Sensitivity analysis

Variables with a range ≥ 1% of base case ICER (comparing each HPV9 combination strategy to HPV4 combination strategy) were listed in descending order (Fig. 3.1–3.3). The HPV4/9 combination strategies were both sensitive to efficacy of HPV4/9 for cervical cancer and genital warts, cost of HPV4/9, the discount rate of effectiveness, utility of warts, age of vaccination, screen coverage, and so on. Threshold analyses showed that when efficacy of HPV9 for genital warts/cervical cancer is smaller than 0.682/0.839, cost of HPV9 more than $567 USD, cost of HPV4 smaller than $283 USD and efficacy of HPV4 for cervical cancer greater than 0.883, ICER exceeds three times of GDP per capita (23,880 USD).

**Fig. 3:**
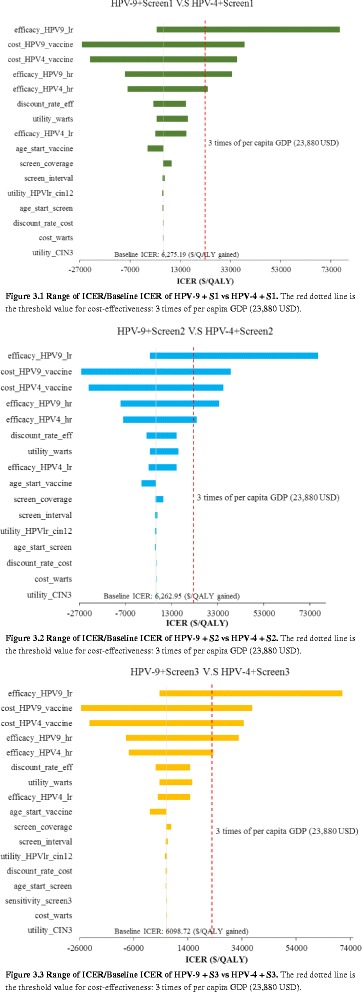
(3.1–3.3) Univariate sensitivity analysis to exmaine variables that impact base case

As HPV2 is the only accessible vaccine on mainland China so far, we also did a one-way sensitivity analysis for HPV2, comparing HPV2 combination strategies to screening alone (Additional file 1: Figure S3.1–S3.3). The results were mainly sensitive to the discount rate of effectiveness, screening coverage, discount rate of cost, utility of warts, CIN 3, age of vaccination, and so on. Since the screening coverage was important and public health meaningful among the results, we performed a threshold analysis of screening coverage from 10% to 100%, comparing it with willingness to pay (three times the GDP per capita of China). The combination of HPV2 with Screening 1 and Screening 2 showed similar values, and when screening coverage was increased to about 61%, these strategies were found to be cost-effective. When screening was increased to 78%, HPV2 combined with Screening 3 was also found to be considered acceptable (ICER doesn’t exceed three times of GDP per capita).

## Discussion

Past population-based screening programs demonstrated the impact of vaccination on reducing cervical cancer mortality and morbidity in both developed and developing countries [45, 46]. Despite the overall decline in its disease burden, cervical cancer still remains the most lethal gynaecological cancer in China. Before HPV vaccines were approved for use on mainland China, the chief strategy for cervical cancer prevention and control was routine screening, targeting women at reproductive age for early detection and treatment. However, well-established evidence indicates that the success of such a program depends on adequate coverage of the population, which has often proved difficult to achieve [47]. As screening alone tends to be insufficient for combating the disease, and ideally primary prevention would be integrated with secondary measures, this study provided a perspective on the potential benefits of innovative vaccination strategies as an adjunct to the current screening program in China. The results of the first economic evaluation of an integrated strategy for the prevention and control of cervical cancer in China presented here will aid in informing policy makers at both the national and local levels.

The results of this study indicated that under baseline coverage of the vaccination and the screening programs, HPV2 vaccine, recently approved in China, is not cost effective either in the single vaccine protocol or in the screening combination strategies; however, the HPV4/9 strategy showed much better cost-effectiveness compared to HPV2 (with ICER of 5400 $/QALY and 5434 $/QALY respectively), and especially the strategy combining HPV9 with screening program showed best health impact on reducing the cervical cancer incidence and mortality rate, compared to that of the HPV2 vaccine combination strategy. These results were consistent with those of studies conducted in the United States [48, 49], which showed that the HPV9 vaccine resulted in a reduction in the incidence of genital warts among different populations [26, 50, 51]. After HPV 16/18, the most common HPV types among Chinese women with either a normal or abnormal cervical diagnosis were HPV 58, 31, and 52, which have been implicated in ICC among Chinese women and have accounted for 9.59% ~ 25.7% of ICC cases [30, 52]. They have now been added to the list of oncogenic types of HPV targeted by the second-generation HPV9 (HPV-31, 33, 45, 52 and 58). Furthermore, the potential benefits of HPV9 include a reduction in the incidence of vulvovaginal, penile, and anal tumours, and paraneoplastic lesions arising in the lower-genital tract [53]. Among boys and male adolescents 55% of anal and penile cancers are caused by the five oncogenic HPV types listed above. HPV9 vaccination not only covers these types specifically but also 68% of all HPV types, and is thus expected to confer significant health benefits in males as well [54]. HPV9 will therefore be an invaluable adjunct to China’s current vaccination program and should be considered seriously by public health policy makers.

Our results highlighted the validity of combining screening and vaccination. Combination strategies using HVP4/9 were cost-effective and able to confer greater health benefits than screening alone. Data from both developed and developing countries thus far have demonstrated that neither secondary prevention, such as screening alone, nor a conventional vaccination program can reduce the incidence of cervical cancers and warts or the disease burden [55]. Our results confirmed, in line with previous economic assessments, that integrated primary prevention (vaccination) and secondary prevention (screening program) were the most effective in terms of cost-effectiveness and health impact [55–57]. At the baseline level the HPV-9 + Screening 1 (Liquid-based cytology test + HPV DNA test) protocol was the most effective, having achieved a reduction of 34.39% in cancer incidence and of 35.95% in cancer deaths.

Vaccination is the primary preventative measure but effective only in those not yet infected by HPV [55]. Moreover, because immunity to HPV is primarily type-specific, the current generation of vaccines against a limited number of HPV types cannot provide complete protection [55]. Therefore, as a preliminary phase in the implementation of combination strategies, the current screening program should be strengthened by improving coverage for the general population [58]. Interestingly the recommended Screening 2 protocol (pap smear cytology test + HPV DNA test) showed almost the same effect as Screening 1 (liquid-based cytology test + HPV DNA test) in terms of reducing the incidence of cancer, HPV infections, and warts. The Screening 3 protocol (VIA), which cost much less and showed great cost-effectiveness, is considered the best choice in medical resource-poor settings.

The different combination strategies examined in this study were flexible enough to be adapted to the wide geographical and socioeconomic diversity found in China according to the varying income levels and availability of medical resources. The Screening 3 or 2 protocols are recommended for low income settings, and if economic conditions permit, the screening plus vaccine strategy, which is likely to achieve have greater prevention impact, is also recommended even if HPV2 vaccine alone is available in mainland China so far.

The sensitivity analysis of HPV9 showed the robustness of its cost-effectiveness. The sensitivity analysis of HPV2, on the other hand, demonstrated that screening coverage crucially influenced the cost-effectiveness of different strategies. This study found that only when screening coverage increased to 60% ~ 70% did the HPV2 and screening combination strategies become economically feasible. In practice, coverage tends to be affected by various factors at both the social and individual level, such as affordability, the socioeconomic status of different regions, the educational level, and adherence of the target population to vaccine protocol recommendations [55]. Regarding the screening program, the current policy tends to be region driven and to focus on high risk populations, leading to a relatively low level of coverage in nonurban areas estimated to comprise approximately 20% of the population [59]. As for vaccination, most developing nations have experienced various challenges including coverage, accessibility, cold-chain transportation, and communication strategies to promote public acceptability [47]. Affordability is essential; high costs without public subsidy may restrict coverage especially among the poor and vulnerable. Educational interventions are also necessary to boost vaccination rates due to relatively poor HPV vaccine awareness among the Chinese [60]. Furthermore, because the vaccines are a relatively new development, the safety and the side effects remain a huge concern globally and affect the public’s perceptions, and consequently the coverage, of any vaccination program [61, 62]. Especially for the newer HPV9 vaccine, more monitoring and surveillance are needed to establish safety and to maintain efficacy and cross-protection against oncogenic HPV types. These uncertainties should be carefully addressed in future studies for their potential impact on public health policy as well.

There were several limitations to this study. First, we did not model the transmission dynamics of HPV nor consider what protection might be conferred by HPV vaccinations against other HPV-related cancers like cervical adenocarcinoma, vulvar carcinoma, and laryngeal papillomatosis due to the lack of data. Consequently, the overall effectiveness of the vaccination program might have been underestimated, thus making the cost-effectiveness of the HPV vaccine appear even more favourable if herd immunity or protection against other diseases existed [63, 64] especially in the case of HPV9, which confers wider coverage compared to the other two vaccines. Moreover, this study did not consider the cross-protective effects of vaccines, multiple HPV infections, and unidentified HPV types, which can influence the effectiveness of vaccines; instead we set a range for vaccine efficacy in our sensitivity analysis to determine the robustness of the model, showing that these variables did not significantly affect the ICERs under the current WTP threshold. In addition, women adhering to previous cervical screening tests may have shown better compliance with subsequent tests [13]. Nevertheless, in the absence of information on local contexts, we chose not to perform individually based modelling, nor did we consider the negative indirect effects.

## Conclusion

We demonstrated that HPV4/9 vaccination for adolescent girls was highly cost-effective in China when integrated into the current screening strategies. Integrated vaccination and screening is recommended. HPV9 vaccination combined with Screening 1 (liquid-based cytology test + HPV DNA test) proved to be the best preventative strategy against cervical cancer and warts.

## Additional file

Additional file 1: Table S1. Transfer probability used in the Markov model. **Figure S1.** Calibration result of age-specific incidence and mortality of cervical cancer. **Figure S2.** Comparing Discounted cost and QALYs with each strategy. S is short for screening; H is short for HPV vaccine. Q is short for QALY. Blue means strategies with screening1, yellow means strategies with screening2 and green screening3. Plots with grey outline means extended dominance. **Figure S3.1.** Spread of ICER/Baseline ICER of HPV-2 + S1 V.S S1. **Figure S3.2.** Spread of ICER/Baseline ICER of HPV-2 + S2 V.S S2. **Figure S3.3.** Spread of ICER/Baseline ICER of HPV-2 + S3 V.S S3. (DOC 288 kb)

## Abbreviations

AIS: Adenocarcinoma In Situ
CIN: Cervical intraepithelial neoplasia
HPV: Human papillomavirus
VIA: Visual inspection with acetic acid
FIGO: International Federation of Gynaecology and Obstetrics
QALY: Quality-adjusted life year
CPI: Consumer price index
ICER: Incremental cost-effectiveness ratio
GDP: Gross domestic product
ICC: Invasive cervical cancer
WTP: Willingness to pay
HPV2, 4, 9: 2-, 4-, 9-valent HPV vaccine
