# Supplementary material for: Cost-effectiveness analysis of different types of human papillomavirus vaccination combined with a cervical cancer screening program in mainland China
Source: BMC Infect Dis. 2017 Jul 18;17:502. doi: 10.1186/s12879-017-2592-5 (PMC5516327; doi:10.1186/s12879-017-2592-5)
Supplement: Additional file 1: Table S1. — Transfer probability used in the Markov model. Figure S1. Calibration result of age-specific incidence and mortality of cervical cancer. Figure S2. Comparing Discounted cost and QALYs with each strategy. S is short for screening; H is short for HPV vaccine. Q is short for QALY. Blue means strategies with screening1, yellow means strategies with screening2 and green screening3. Plots with grey outline means extended dominance. Figure S3.1. Spread of ICER/Baseline ICER of HPV-2 + S1 V.S S1. Figure S3.2. Spread of ICER/Baseline ICER of HPV-2 + S2 V.S S2. Figure S3.3. Spread of ICER/Baseline ICER of HPV-2 + S3 V.S S3. (DOC 288 kb) [file 12879_2017_2592_MOESM1_ESM.doc]

**Additional file 1**

**Table S1: Transfer probability used in the Markov model**

| Variables | Age | Transfer probability |
| --- | --- | --- |
| **Health to** |  |  |
| High risk HPV infection | 15- | 0.0200 |
|  | 18- | 0.1263 |
|  | 24 | 0.1263 |
|  | 25- | 0.1176 |
|  | 35- | 0.1351 |
|  | 45- | 0.1202 |
|  | 65- | 0 |
| Low risk HPV infection |  | 0.0315 |
| **High risk HPV infection to** |  |  |
| CIN1 | 15- | 0.16 |
|  | 25- | 0.09 |
|  | 55- | 0.07 |
| CIN2 | 15- | 0.05 |
|  | 25- | 0.02 |
|  | 35- | 0.01 |
|  | 55- | 0.008 |
| Health | 15- | 0.6 |
|  | 20- | 0.6 |
|  | 25- | 0.35 |
|  | 30- | 0.3 |
|  | 81 | 0 |
| **High risk of CIN1 to** |  |  |
| CIN2 | 15- | 0.026 |
|  | 20- | 0.026 |
|  | 25- | 0.03 |
|  | 45- | 0.04 |
| CIN3 | 15- | 0.026 |
|  | 25- | 0.03 |
|  | 40- | 0.0331 |
| High risk of HPV infection |  | 0.23 |
| Health |  | 0.23 |
| **High risk of CIN2 to** |  |  |
| CIN3 | 15- | 0.1 |
|  | 25- | 0.15 |
|  | 35- | 0.18 |
|  | 45- | 0.2 |
|  | 55- | 0.22 |
|  | 65- | 0.24 |
|  | 75- | 0.26 |
| High risk of HPV infection |  | 0.035 |
| Health |  | 0.315 |
| **High risk of CIN3 to** |  |  |
| CIN1 | 15- | 0.07 |
|  | 35- | 0.03 |
|  | 60- | 0.01 |
| CIN2 | 15- | 0.05 |
|  | 40- | 0.02 |
|  | 50- | 0.015 |
|  | 60- | 0.01 |
|  | 75- | 0.005 |
| Cancer |  | 0.0105 |
| **Low risk HPV infection to** |  |  |
| Low risk of CIN1/2 |  | 0.104 |
| Genital warts |  | 0.57 |
| Low risk of CIN1/2 to Health/ infected |  | 0.552 |
| Genital warts to Health/ infected |  | 0.875 |
| **Cancer stage** |  |  |
| Localized FIGO I-IIA (48.8%) | 30- | 0.032 |
|  | 40- | 0.035 |
|  | 60- | 0.039 |
|  | 80- | 0.205 |
| Regional FIGO IIB-IVA (31.5%) | 30- | 0.067 |
|  | 40- | 0.085 |
|  | 60- | 0.091 |
|  | 80- | 0.29 |
| Metastatic FIGO IVB (19.7%) | 30- | 0.067 |
|  | 40- | 0.085 |
|  | 60- | 0.091 |
|  | 80- | 0.29 |

**Reference:**

[1] Canfell, K., Barnabas, R., Patnick, J., Beral, V., The predicted effect of changes in cervical screening practice in the UK: results from a modelling study. Br J Cancer, 2004. 91(3): p. 530-6.

[2] Li, D.Y., Health economic evaluation and economic feasibility research of cervical cancer screening in rural areas. 2010, Medical school of Dalian. Master degree.

[3] Shi, J.F., Cost-effectiveness on various modalities of cervical cancer screening in rural China. 2009, Chinese Academy Of Medical Sciences & Peking Union Medical College. Doctor degree.

[4] Mennini, F.S., Giorgi-Rossi, P.F., Palazzo, F., Largeron, N., Health and economic impact associated with a quadrivalent HPV vaccine in Italy. Gynecol Oncol, 2009. 112(2): p. 370-6.

[5] Quinn, M. A., Benedet, J. L., Odicino, F., Maisonneuve, P., Beller, U., Creasman, W. T., Heintz, APM., Ngan, HYS., Pecorelli, S., Carcinoma of the cervix uteri. FIGO 26th Annual Report on the Results of Treatment in Gynecological Cancer. Int J Gynaecol Obstet, 2006. 95 Suppl 1: p. S43-103.

[6] Siebert, U., Sroczynski, G., Hillemanns, P., Engel, J., Stabenow, R., Stegmaier, C., Voigt, K., Gibis, B., Holzel, D., Goldie, S. J., The German cervical cancer screening model: development and validation of a decision-analytic model for cervical cancer screening in Germany. Eur J Public Health, 2006. 16(2): p. 185-92.

[7] Termrungruanglert, W., Havanond, P., Khemapech, N., Lertmaharit, S., Pongpanich, S., Khorprasert, C., Taneepanichskul, S., Cost and effectiveness evaluation of prophylactic HPV vaccine in developing countries. Value Health, 2012. 15(1 Suppl): p. S29-34.


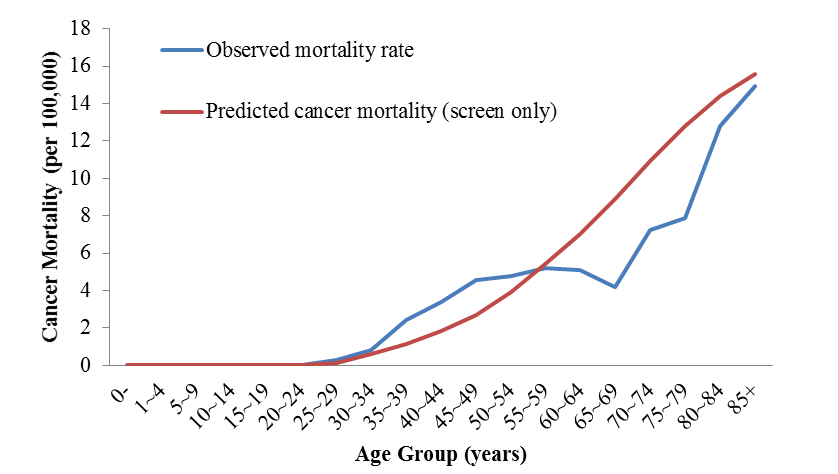


**Figure S1. Calibration result of age-specific incidence and mortality of cervical cancer**

**
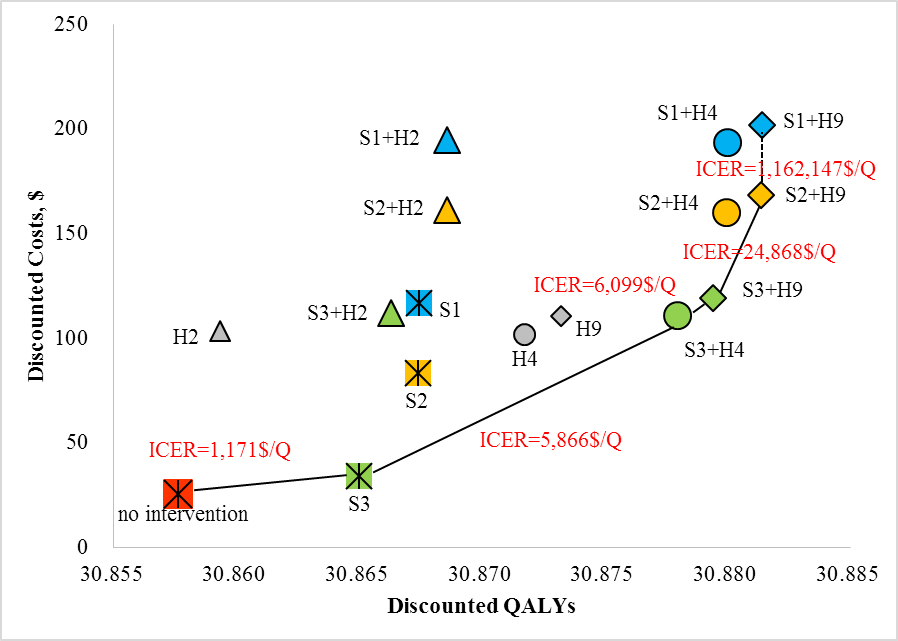
Figure S2 Comparing Discounted cost and QALYs with each strategy** S is short for screening; H is short for HPV vaccine. Q is short for QALY. Blue means strategies with screening1, yellow means strategies with screening2 and green screening3. Plots with grey outline means extended dominance.


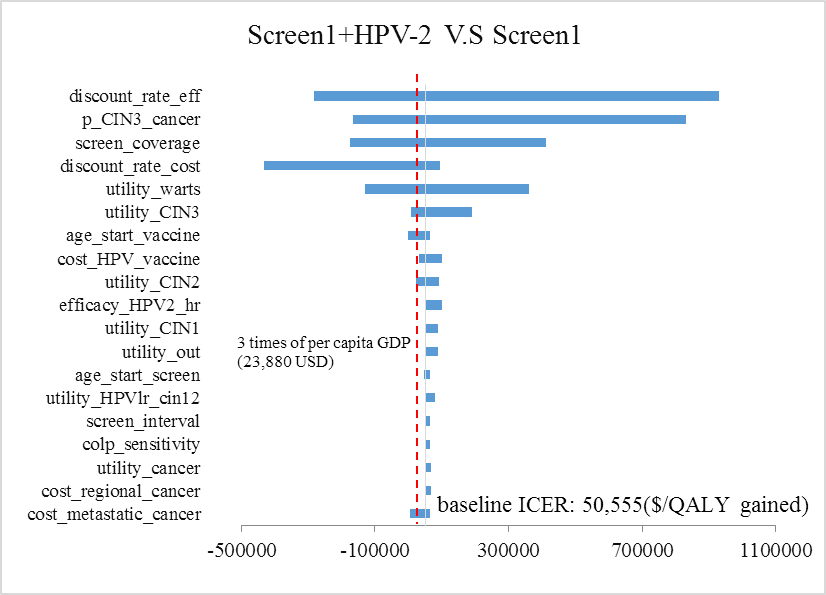


**Figure S3.1 Spread of ICER/Baseline ICER of** **HPV-2 +S1 V.S S1**


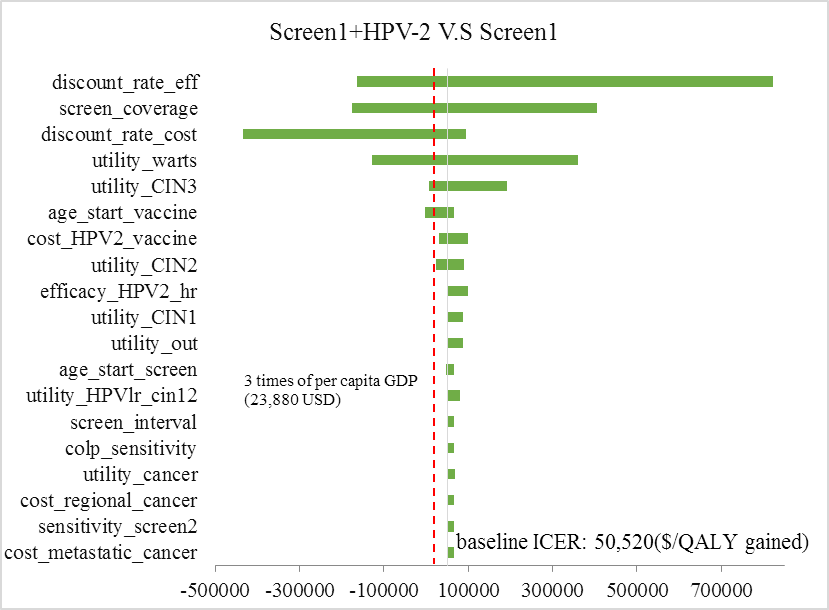


**Figure S3.2 Spread of ICER/Baseline ICER of HPV-2 +S2 V.S S2**


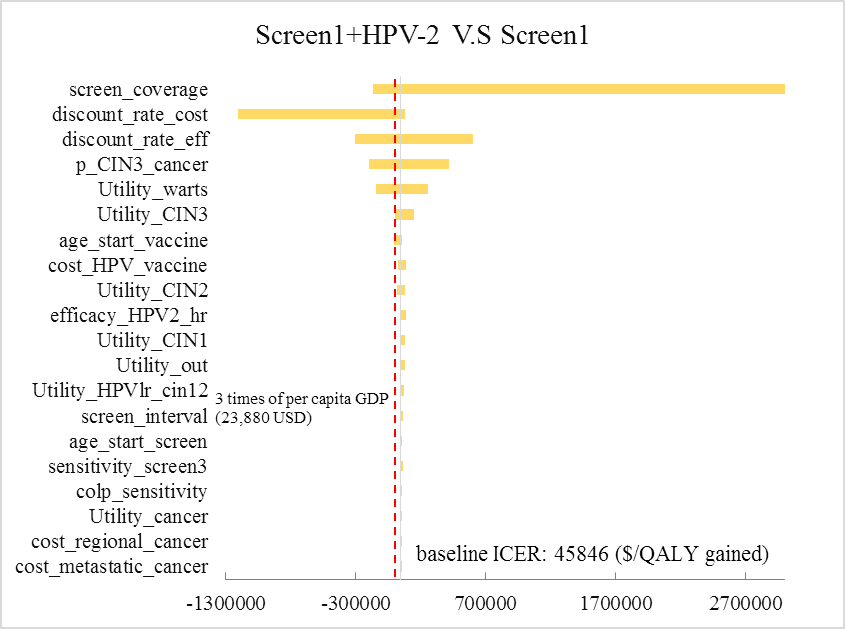


**Figure S3.3 Spread of ICER/Baseline ICER of HPV-2 +S3 V.S S3**
